# Cognitive decline in post-COVID-19 syndrome does not correspond with persisting neuronal or astrocytic damage

**DOI:** 10.1038/s41598-024-55881-1

**Published:** 2024-03-04

**Authors:** Fabian Boesl, Yasemin Goereci, Finja Schweitzer, Carsten Finke, Ann-Katrin Schild, Stefan Bittner, Falk Steffen, Maria Schröder, Anneke Quitschau, Josephine Heine, Clemens Warnke, Christiana Franke

**Affiliations:** 1 Department of Neurology, Charité-University Medicine Berlin, Berlin, Corporate Member of Freie Universität Berlin, Humboldt-Universität zu Berlin, Berlin Institute of Health, Hindenburgdamm 30, 12203 Berlin, Germany; 2 Department of Neurology, Faculty of Medicine and University Hospital Cologne, University of Cologne, Cologne, Germany; 3 Department of Psychiatry, Faculty of Medicine and University Hospital Cologne, University of Cologne, Cologne, Germany; 4 Department of Neurology, Focus Program Translational Neuroscience (FTN) and Immunotherapy (FZI), University Medical Center of the Johannes Gutenberg University Mainz, Rhine-Main Neuroscience Network (rmn2), Mainz, Germany

**Keywords:** Neurological disorders, Biomarkers

## Abstract

Cognitive impairment is the most frequent symptom reported in post-COVID-19 syndrome (PCS). Aetiology of cognitive impairment in PCS is still to be determined. Neurofilament light chain (NfL) and glial fibrillary acidic protein (GFAP) are increased in acute COVID-19. Their role as biomarkers in other neurological disorders is under debate. We analysed serum levels of NfL and GFAP as markers for neuronal and astrocytic damage in 53 patients presenting to a PCS Neurology outpatient clinic. Only individuals with self-reported cognitive complaints were included. In these individuals, cognitive complaints were further assessed by comprehensive neuropsychological assessment (NPA). Patients were categorized into subgroups of subjective cognitive decline, single domain impairment, or multi-domain impairment. Serum NfL was in normal range, however an increase of serum GFAP was detected in 4% of patients. Serum NfL and GFAP levels correlated with each other, even when adjusting for patient age (r = 0.347, p = 0.012). NPA showed deficits in 70%; 40% showing impairment in several tested domains. No significant differences were found between serum NfL- and GFAP-levels comparing patients with subjective cognitive decline, single domain impairment, or multi-domain impairment. Persistent neuronal or astrocytic damage did not correlate with cognitive impairment in PCS.

## Introduction

Symptoms in post-COVID-19 syndrome (PCS), also referred to as post-acute sequelae of COVID-19, are manifold and include neurological and neuropsychiatric symptoms^1^. One of the most frequently reported symptoms in patients with PCS are cognitive complaints, which can be evaluated by neuropsychological assessment (NPA)^2–5^. To date, aetiology of neurological symptoms in PCS is still unknown.

Cerebrospinal fluid (CSF) and blood neurofilament light chain (NfL) have been used as a biomarker for neuronal axonal damage for several neurological disorders^6^. Elevated glial fibrillary acidic protein (GFAP) levels have been monitored to assess astrocytic activation and damage^7^. With regard to COVID-19, several studies showed an increase of CSF and blood NfL and GFAP in moderate and severe cases of acute disease, even without major central-nervous pathologies^8–15^. COVID-19 severity and mortality have been correlated with increased NfL and GFAP levels^8–15^.

Furthermore, elevation of those markers in hospitalized COVID-19 patients have been associated with long-term neurological symptoms including cognitive impairment^16^. In contrast, in a longitudinal analysis of blood GFAP- and NfL-levels of COVID-19 patients, normal levels were noted 6 months after acute SARS-CoV-2 infection following an initial peak, although half of the patients reported persistent neurological symptoms^17^.

In this study, we analysed serum NfL and GFAP levels of patients presenting to a PCS Neurology outpatient clinic, predominantly reporting of cognitive complaints. A cognitive screening and a NPA were performed to evaluate reported cognitive deficits.

## Results

A total of 53 patients (Berlin, n = 18; Cologne, n = 35) were included in this study. 62% of patients were female. Mean age was 45.1 years (24–71 years). Median time between acute SARS-CoV-2 infection and analysis of serum NfL and GFAP and NPA was 232 days. Median time between NPA and blood sampling was 20 days. The majority of patients had a mild COVID-19 course (91%) according to the WHO Clinical Progression Scale^18^. MoCA results were abnormal (< 26) in 30% of the patients, even though all patients reported cognitive complaints. The most frequent concomitant neurological or neuropsychiatric PCS symptoms were fatigue (75%), sleep disorders (42%) and depression (34%). Though fatigue was the second most prevalent symptom, only two patients (4%) fulfilled diagnostic criteria for Myalgic encephalomyelitis/chronic fatigue syndrome (ME/CFS) according to the 2003 Canadian Consensus Criteria^19^. Patients’ characteristics and PCS symptoms are shown in Table 1. PCS symptoms were additionally plotted as an Upset Plot to show intersections (Supplemental Fig. S1). Available cranial MRI scans (n = 43) were mostly lacking structural pathologies (n = 35, 81%). Detected pathologies were an aneurysm of the medial cerebral artery (n = 1); occipital microbleeds (n = 1); unspecific white matter lesions (n = 4); slightly prominent parietal sulci (n = 1) and leukoaraiosis stable compared to scans from before COVID-19 (n = 1).

**Table 1:** Patients' characteristics and post-COVID-19 symptoms.

Number of patients	53	
Berlin	18	
Cologne	35	
Age (mean; range in years)	45.1	24–71
Gender (n, %)		
Female	33	62
Male	20	38
Non-binary	0	0
Course COVID-19 (n, %)		
Mild	48	91
Moderate	2	4
Severe	3	6
Time between COVID-19 and blood sample (median, range in days)	232	100–598
Time between COVID-19 and neuropsychological assessment (median, range in days)	232	109–467
Time between neuropsychological assessment and blood sample (median, range in days)	20	0–226
Symptoms (n, %)		
Cognitive complaints	53	100
Fatigue	40	75
Sleep disorder	22	42
Depression	18	34
Headache	17	32
Myalgia	12	23
Hyposmia/hypogeusia	11	21
Vertigo	8	15
Sensibility dysfunction	4	8
Cranial MRI scans (n, %)	43	81
Pathological findings	8/43	19
MoCA		
Median, IQR	27	25–29
< 26 (abnormal; n, %))	16	30
≥ 26 (normal; n, %)	37	70
Neuropsychological assessment (n, %)		
Subjective cognitive decline	16	30
Single-domain impairment	16	30
Multi-domain impairment	21	40
GFAP (pg/ml; median, IQR)	89.7	67.0–107.6
NfL (pg/ml; median, IQR)	7.4	6.0–10.0

Demographics, patients’ characteristics, post-COVID-19 symptoms, results of Montreal Cognitive Assessment (MoCA) and neuropsychological assessment (NPA) and results of neurofilament light chain (NfL) and glial fibrillary acidic protein (GFAP) measurement.

**Figure 1:**
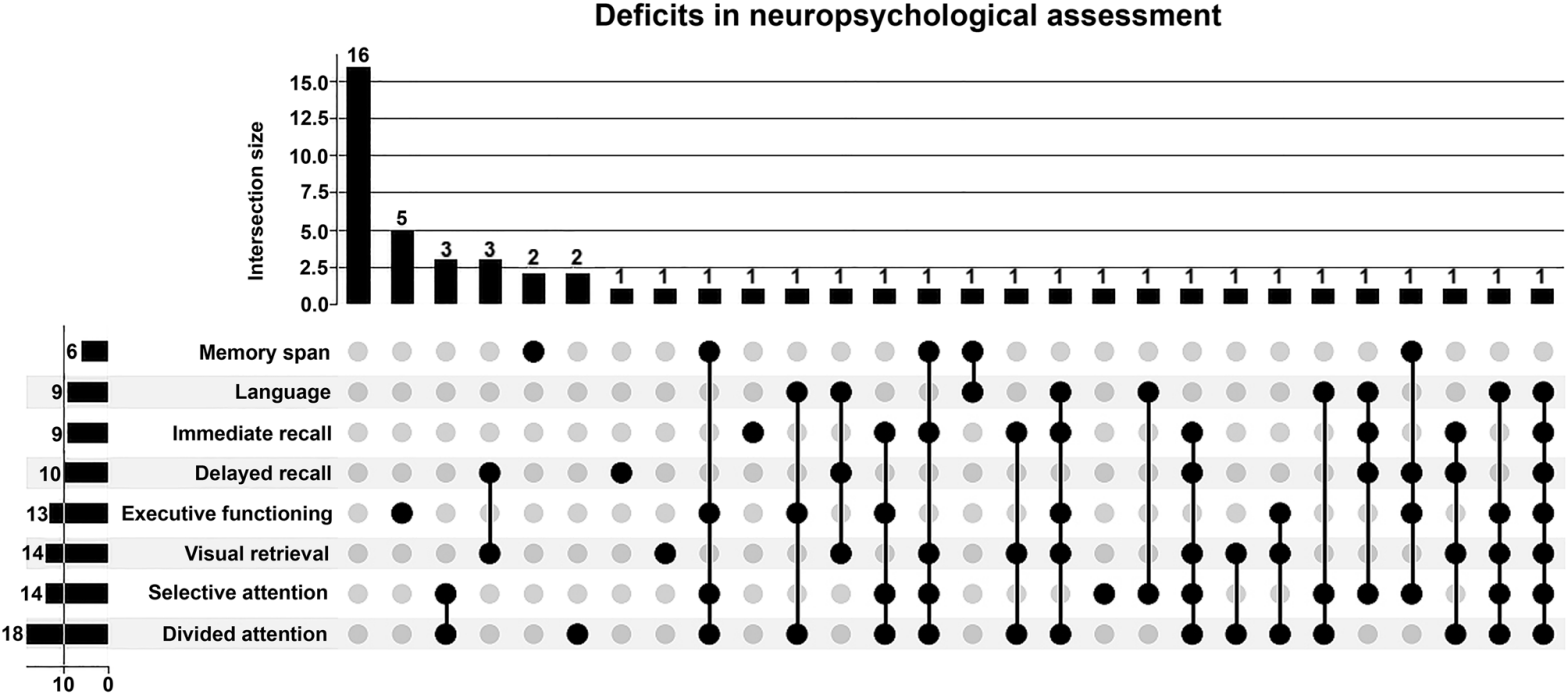
Deficits in neuropsychological assessment and their intersections plotted as an Upset Plot.

NPA confirmed cognitive deficits in 37 patients (70%). 16 patients (30%) had deficits in only one domain, while 21 (40%) had deficits in two or more domains. Deficits were detected most frequently in divided attention (n = 18, 34%), followed by selective attention (n = 14, 26%) and visual retrieval (n = 14, 26%). A predominant phenotype of cognitive deficits was not detectable, as shown in an Upset Plot of deficits in NPA and their intersections (Fig. 1). MoCA total scores correlated with the NPA category (r = − 0.325, p = 0.017).

Median value of serum NfL was 7.4 pg/ml (interquartile range 6.0–10.0 pg/ml), and 89.7 pg/ml for GFAP (interquartile range 67.0–107.6 pg/ml). Considering established age-specific reference levels for serum NfL and GFAP (Supplemental Tables S1 and S2)^20,21^, all detected NfL levels were within the upper limit of normal and elevated serum GFAP levels were found in only two individuals (4%).

**Figure 2:**
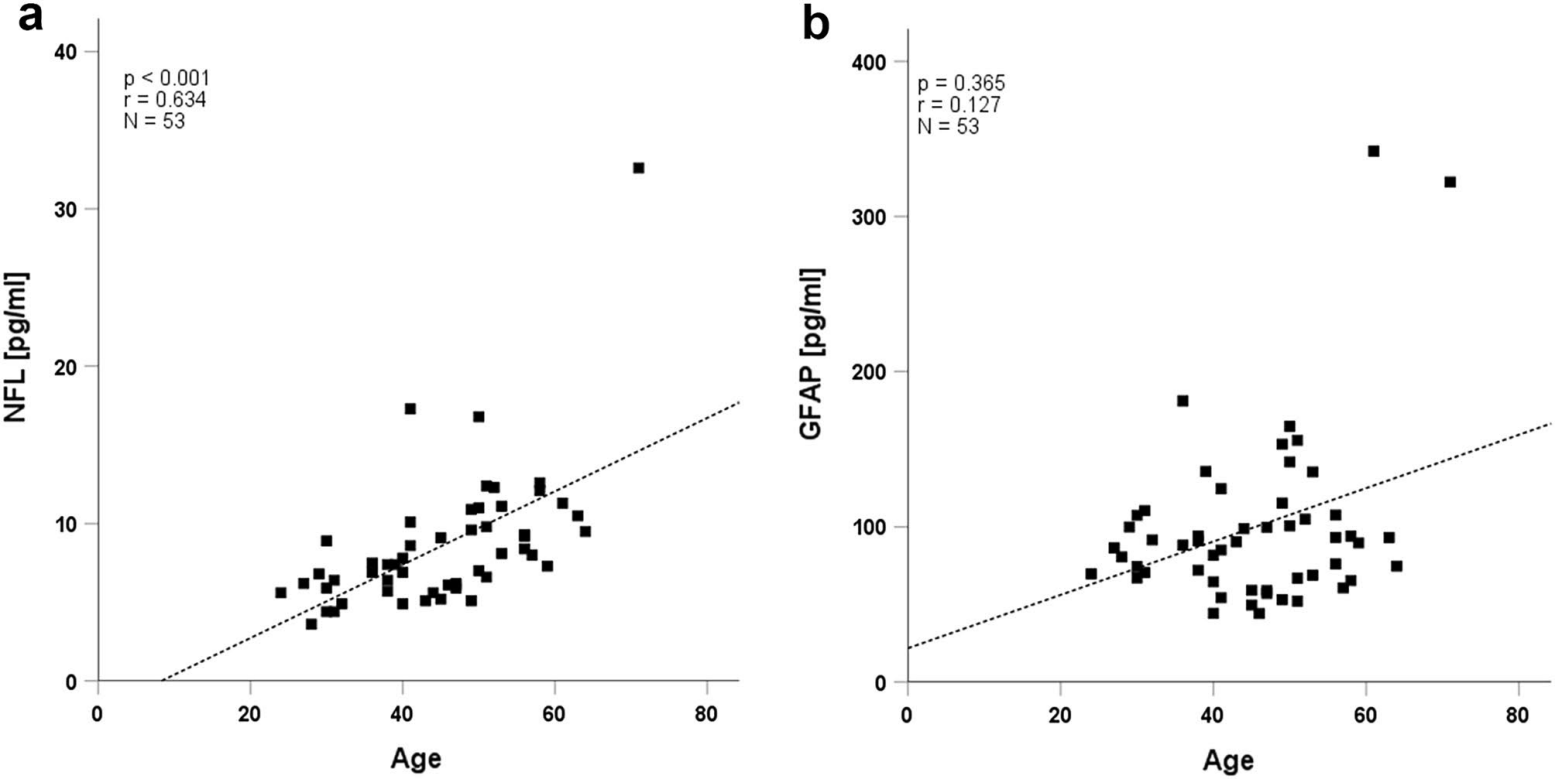
Correlation plot of neurofilament light chain (NfL) (**a**) or glial fibrillary acidic protein (GFAP) (**b**) with age.

**Figure 3:**
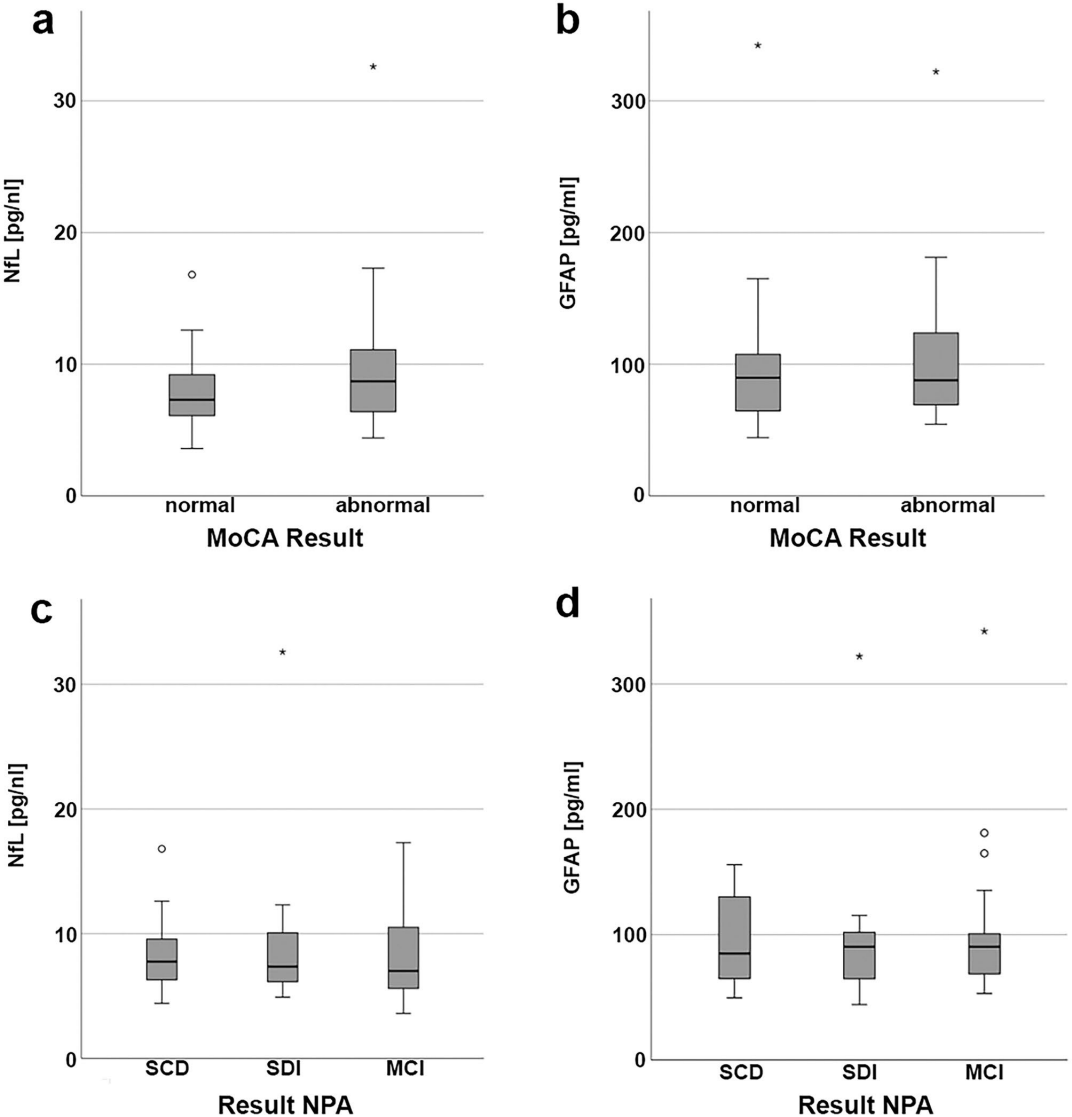
Boxplots of levels of neurofilament light chain (NfL) or glial fibrillary acidic protein (GFAP) in different cognitive subgroups. (**a**) Levels of NfL in the groups of normal and abnormal score in the Montreal Cognitive Assessment (MoCA). (**b**) Levels of GFAP in the groups of normal and abnormal score in the MoCA. (**c**) Levels of NfL in the groups of subjective cognitive decline (SCD), single domain impairment (SDI) and multi-domain impairment (MDI) according to neuropsychological assessment (NPA). (**d**) Levels of GFAP in the groups of SCD, SDI and MDI according to NPA.

Serum NfL levels correlated with age as expected (r = 0.634, p < 0.001; Fig. 2a), while GFAP did not (r = 0.127, p = 0.365; Fig. 2b). Levels of NfL and GFAP correlated with each other, also when adjusting for age (r = 0.347, p = 0.012). NfL and GFAP levels did not differ between patients with normal and abnormal MoCA scores (p = 0.20 for NfL, p = 0.45 for GFAP; Fig. 3a,b) or between patients with SCD, SDI or MDI (p = 0.680 for NfL, p = 0.895 for GFAP; Fig. 3c,d).

## Discussion

Elevation of GFAP and NfL has been reported in the acute phase of COVID-19, particularly in severe cases^10,14,15^. A Chinese cohort study showed that severe COVID-19 is associated with long term cognitive impairment 12 months after acute disease^22^. In a cohort of hospitalized COVID-19 patients from the UK, significantly higher values of NfL and GFAP were measured compared to controls in the acute phase^23^. Patients who developed neurological complications such as cerebrovascular incidents still presented elevated NfL values in the late convalescent phase, which was defined as more than 6 weeks after first positive SARS-CoV-2 PCR^23^. Cognitive impairment after severe COVID-19 might be attributed to neuronal damage in the acute phase. Whether these deficits and their aetiology differ from cognitive impairment in post-intensive care syndrome (PICS)^24^ is still to be determined and needs to be the subject of further studies.

Our data does not support the hypothesis of persisting neuronal or astrocytic damage as the leading cause of cognitive decline in PCS. This is in line with previous reports showing normalization of GFAP and NfL levels 6 months after acute COVID-19^17^ and normal blood NfL- and GFAP levels in patients with persisting headache in PCS^25^. As such, serum NfL and GFAP appear not to be sensitive biomarkers for cognitive impairment in PCS. Interestingly, recent findings in a Spanish cohort of PCS patients with subjective cognitive complaints revealed reduced hippocampal grey matter volume and elevated NfL and GFAP levels compared to controls but did not apply age-adjusted reference values^26^. Implication for clinical assessment as biomarker usage is questionable to date.

In our cohort, most patients had a mild COVID-19 course. Here, other pathomechanisms than those discussed after severe COVID-19 are of potential relevance and under debate^27^. Previous findings suggest that a persistent central nervous system infection with SARS-CoV-2 may not be the cause of neurological or neuropsychiatric symptoms in PCS^28^. Patients with cognitive impairment in PCS showed higher CCL11/eotaxin-1 plasma values than patients with non-cognitive PCS symptoms, a cytokine that leads to specific reactivity of hippocampal microglia and impaired hippocampal neurogenesis in mice after systemic application^29^. Neuroinflammation and microglial reactivity and their role in cognitive impairment in PCS should be further evaluated. Furthermore, the role of autoimmunity in cognitive decline in PCS is of concern, since brain-binding autoantibodies can be found in CSF of patients with PCS and are associated with cognitive impairment^30^.

Although—as predefined per study inclusion criteria—all patients in our study reported subjective cognitive complaints, less than a third had abnormal MoCA values. Via comprehensive NPA, deficits were detectable in almost 70%, with approximately 40% having deficits in several neuropsychological domains. Interestingly, 30% did not show any deficits including NPA. This could be due to higher premorbid cognitive capacities and therefore higher premorbid test scores of those patients, with their current test scores still ranging within normalised means. Since neuropsychological data of our cohort prior to COVID-19 is lacking, this remains hypothetical. The discrepancy between subjective cognitive complaints and objective cognitive impairment in NPA has been previously reported for PCS^31^. A PCS specific phenotype restricted to specific neuropsychological deficits could not be determined by our study.

Frequent concomitant symptoms were fatigue and depressive symptoms since COVID-19. ME/CFS is commonly associated with cognitive impairment and also presenting with a heterogeneous profile in NPA^32^. Cognitive dysfunction is also a leading symptom in depression. In depressive patients, NPA shows deficits in several domains, even after remission^33^. Whether cognitive impairment in PCS is a singular entity or the result of other, potentially multifactorial, determinants, needs to be the subject of further studies.

The strengths of our study are the combination of biomarker analyses with a comprehensive neuropsychological assessment as well as the inclusion of patients at two centres. To minimize bias of our data, inclusion and exclusion criteria were applied to analyse alterations in NfL, GFAP levels and results in NPA most likely caused by post-COVID-19 syndrome and not by other disorders. Limitations of our study are the lack of sequential biomarker and neuropsychological assessments. While there is data showing improvement of cognitive deficits over time, other cohorts found a persisting decrease of processing speed over a 6-month period^34,35^.To provide further longitudinal neuropsychological testing in PCS, some participants of our cohort from the study centre in Cologne were evaluated in neuropsychological follow ups. Due to ongoing analyses, results of these follow ups will be published separately. Another limitation is the assessment of only two biomarkers of possible interest. NfL and GFAP were selected in this study due to the reported elevation in acute COVID-19. Not evaluated in this study were several other cytokines, chemokines and hormones such as tumor necrosis factor, CXCL9 and cortisol, which have been reported to be altered in PCS and could potentially serve as biomarkers for PCS^36–38^.

## Methods

### Study population

Patients (n = 198) presenting to the PCS Neurology outpatient clinics in Berlin and Cologne were screened by a physician specialized in Neurology or Psychiatry for study eligibility. If available, cranial MRI scans, which were performed due to cognitive complaints after COVID-19, were evaluated for causative structural cerebral changes. Only patients fulfilling the PCS criteria by the WHO Delphi consensus^39^ and with a confirmed diagnosis of SARS-CoV-2 infection (either positive PCR testing for SARS-CoV-2-RNA or positive testing for SARS-CoV-2 antibodies prior to vaccination), and who predominantly reported of cognitive complaints following COVID-19 were included in this study. Patients with pre-existing neurological or psychiatric diagnosis, especially prior affective disorders, were excluded, resulting in 53 patients participating. All participants gave informed written consent. This study was approved by the ethics committee of Charité-Universitätsmedizin Berlin (EA2/066/20) and the ethics committee of the University of Cologne (20–1501). The study was conducted in accordance with the declaration of Helsinki.

### Data collection

#### Neuropsychological assessment

The Montreal Cognitive Assessment Scale (MoCA) was performed to screen for cognitive impairment^40^. Further NPA comprised well-validated tests covering the neuropsychological domains of attention, executive functioning, verbal memory, language and visual retrieval (Table 2). Selective attention was evaluated via the Trail Making Test (TMT) A or the Test of Attentional Performance (TAP) tonic alertness^41,42^. Divided attention was evaluated via TMT B or omissions in the TAP divided alertness testing^41,42^. Working memory components of executive function was analysed by testing of the digit span backwards (DSB) of the Wechsler Memory Scale—Revised Manual (WMS-R)^43^. For verbal memory, the German version of the Auditory Verbal Learning Test (Verbaler Lern- und Merkfähigkeitstest; VLMT) or the word list learning of the Consortium to Establish a Registry for Alzheimer’s Disease neuropsychological test battery (CERAD) was assessed^44–46^. Memory span (VLMT—first trial [D1]; CERAD—Word List Learning—first trial), immediate recall (VLMT—Trial 1 to 5 [∑D1-5]; CERAD—Word List Learning Total) and delayed recall (VLMT—Trial 7 [D7]; CERAD—Word List Recall) were evaluated^44–46^. Language was assessed via evaluation of semantic verbal fluency as part of the Regensburger Wortflüssigkeitstest (RWT), a German version of the Controlled Oral Word Association test or the CERAD battery^45–47^. Visual retrieval was evaluated either by the Rey-Osterrieth Complex-Figure-Test (ROCF)—delayed recall, by the Wechsler Memory Scale (WMS) IV subtest visual memory II or by the CERAD constructional praxis—delayed recall^45,46,48,49^. Test results were interpreted as impaired, if the achieved results were less than one standard deviation below test-specific age-, educational level–, and sex-adjusted normalised means. Results of NPA were categorized as subjective cognitive decline (SCD), if NPA showed no impaired results, since all patients self-reported cognitive complaints; single-domain impairment (SDI), if only one of the analysed domains was impaired; or multi-domain impairment (MDI), if two or more domains were concerned.

**Table 2:** Examined cognitive domains and analysed neuropsychological tests.

Domain	Subdomain	Analysed test	n
Attention	Selective attention	TMT A	47
		TAP tonic alertness	5
	Divided attention	TMT B	48
		TAP divided alertness—omissions	5
Executive functioning		WMS-R—Digit span backwards	53
Verbal memory	Memory span	VLMT—D 1	40
		CERAD—Word List Learning—first trial	13
	Immediate recall	VLMT—∑D1-5	40
		CERAD—Word List Learning Total	13
	Delayed recall	VLMT—D7	40
		CERAD—Word List Recall	13
Language		RWT—semantic verbal fluency (animals)	38
		CERAD—semantic verbal fluency (animals)	13
Visual retrieval		ROCF—delayed recall	17
		WMS IV—visual memory II	23
		CERAD constructional praxis—delayed recall	13

*TMT* trail making test, *TAP* test of attentional performance, *WMS-R* Wechsler Memory Scale-Revised Manual, *VLMT* Verbaler Lern- und Merkfähigkeitstest, *CERAD* consortium to establish a registry for Alzheimer’s disease neuropsychological test battery, *RWT* Regensburger Wortflüssigkeitstest, *ROCF* Rey-Osterrieth complex-figure-test, *WMS IV* Wechsler Memory Scale.

#### GFAP and NfL analysis

Serum NfL and GFAP levels were measured in duplicates using the single molecule array HD-X analyzer (Quanterix, Boston, MA) and the NF-light Advantage Kit and GFAP Discovery Kit according to the manufacturer’s protocol.

### Data analysis

Statistics were computed using SPSS (IBM Corp. 2013 SPSS Statistics for Windows, version 29.0). Spearman’s rho was performed for bivariate correlation testing. Partial rank correlation was used for non-parametric partial correlation testing. Welch’s t-Test was used to analyse differences in NfL or GFAP levels in patients with normal and abnormal MoCA scores. One way ANOVA was used to analyse differences in NfL or GFAP levels in patient subgroups. An α-level of 0.05 was chosen for determination of statistical significance. Upset Plots were created with Python^50^.

### Supplementary Information

Supplementary Information.

## Data Availability

The data that support the findings of this study are available from the corresponding author upon reasonable request.
