# Supplementary material for: Cognitive decline in post-COVID-19 syndrome does not correspond with persisting neuronal or astrocytic damage
Source: Sci Rep. 2024 Mar 4;14:5326. doi: 10.1038/s41598-024-55881-1 (PMC10912552; doi:10.1038/s41598-024-55881-1)

Supplemental Table S1

Age-specific upper reference limit for serum NfL

Age (years)	Upper reference limit (pg/ml)
<= 20	<= 7.4
21 – 30	<= 9.9
31 – 40	<= 13.1
41 – 50	<= 17.5
51 – 60	<= 23.3
61 – 70	<= 30.9
71 – 80	<= 41.3
81 – 90	<= 54.9

Supplemental Table S2

Age-specific upper reference limit for serum GFAP

Age (years)	Upper reference limit (pg/ml)
20 – 39	<= 136
40 – 64	<= 242
65 – 90	<= 438

Supplemental Figure S1

Reported Post-COVID-19 symptoms and their intersections plotted as an Upset Plot.

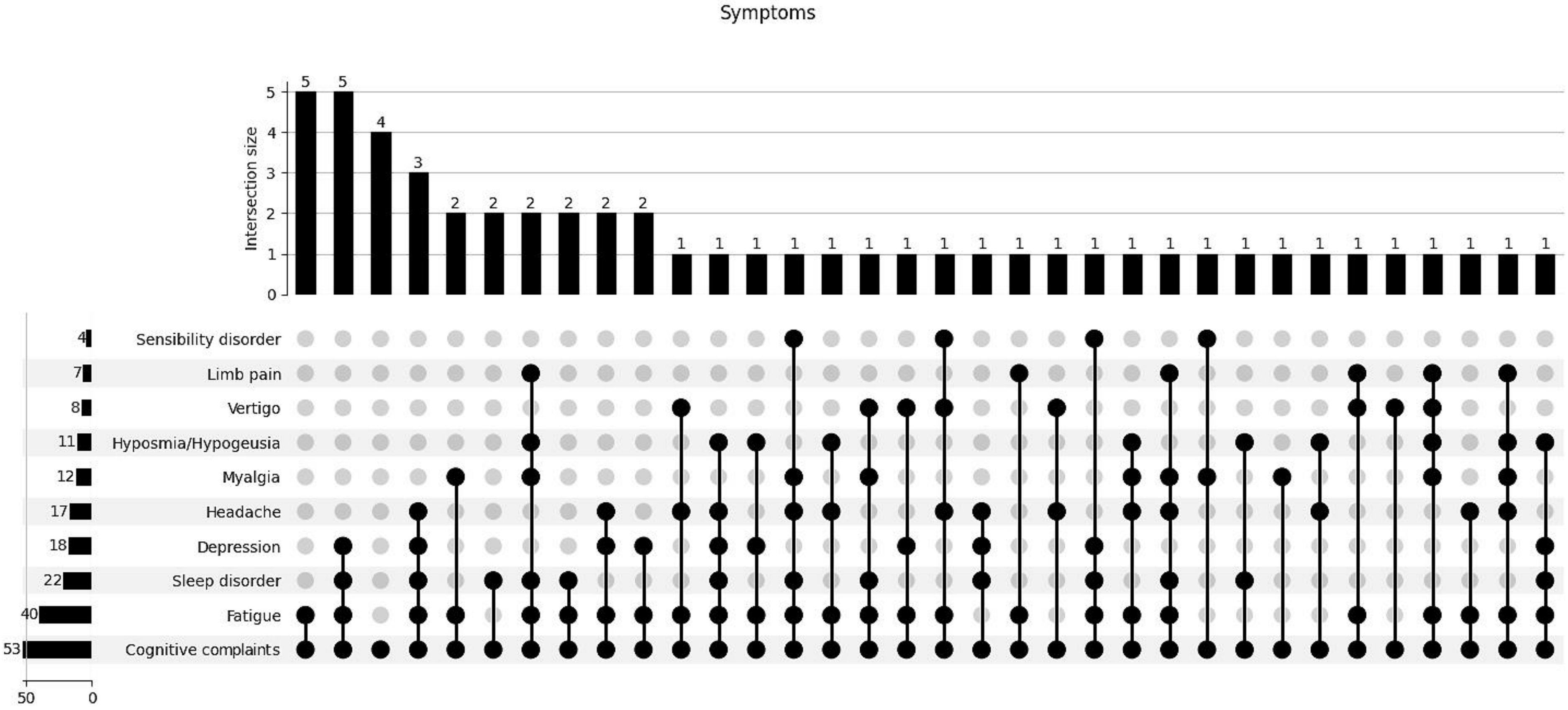

Supplement: Supplementary file 1 — Supplementary Information. [file 41598_2024_55881_MOESM1_ESM.pdf]
